# Climate Change and Health under the Shared Socioeconomic Pathway Framework

**DOI:** 10.3390/ijerph15010003

**Published:** 2017-12-21

**Authors:** Samuel Sellers, Kristie L. Ebi

**Affiliations:** Center for Health and the Global Environment, University of Washington, Seattle, WA 98105, USA; krisebi@uw.edu

**Keywords:** health systems, climate adaptation, health workers, health infrastructure

## Abstract

A growing body of literature addresses how climate change is likely to have substantial and generally adverse effects on population health and health systems around the world. These effects are likely to vary within and between countries and, importantly, will vary depending on different socioeconomic development patterns. Transitioning to a more resilient and sustainable world to prepare for and manage the effects of climate change is likely to result in better health outcomes. Sustained fossil fuel development will likely result in continued high burdens of preventable conditions, such as undernutrition, malaria, and diarrheal diseases. Using a new set of socioeconomic development trajectories, the Shared Socioeconomic Pathways (SSPs), along with the World Health Organization’s Operational Framework for Building Climate Resilient Health Systems, we extend existing storylines to illustrate how various aspects of health systems are likely to be affected under each SSP. We also discuss the implications of our findings on how the burden of mortality and the achievement of health-related Sustainable Development Goal targets are likely to vary under different SSPs.

## 1. Introduction

Climate change is projected to affect health outcomes in a variety of ways, including increases in the frequency and intensity of extreme temperatures and storm events, expansion of the geographic ranges for disease vectors such as mosquitoes, and changes in crop yields that affect nutritional intake [1]. Climate change will also likely affect the ability of health systems to function effectively as a result of changes in demands for services, the effects of climate variability on health infrastructure, and increased costs to provide services in a world impacted by climate change. However, these effects will vary locally as the impacts of climate change are not uniform. Rather, there are a variety of factors that will likely influence outcomes, including a country’s physical location and exposure to climate shocks, current and projected levels of socioeconomic development, as well as healthcare capacity and spending, and finally, changes in the capacity to cope with climate shocks in the future [2]. It is this final factor—changes in the capacity to plan, provide, and finance effective healthcare services in a world impacted by climate change—that we focus on in this paper. Thus, we discuss how different development trajectories may affect health systems, as well as briefly highlight the implications of these effects on changes in causes of mortality and the Sustainable Development Goals (SDGs). 

To date, there has been limited work exploring the implications of climate change on health outcomes under different Shared Socioeconomic Pathways (SSPs) [3]. We expand upon earlier narratives describing the relationship of climate change to health systems—defined by the World Health Organization as the “organizations, people and actions whose primary intent is to promote, restore or maintain health” [4]. For this purpose, we adopt two frameworks. First, we use the SSP framework, which was developed to describe potential future socioeconomic development trajectories in a world impacted by climate change [5]. The SSP framework is a successor to the earlier Special Report on Emissions Scenarios that are increasingly dated [6]. The SSPs are designed to be combined with greenhouse gas emission pathways (representative concentration pathways, RCPs), in a scenario matrix [5,7,8]. This design allows researchers to test different combinations of socioeconomic development and emissions, producing a range of quantitative estimates of risk. Researchers have used this design to quantify aspects of the pathways, including GDP [9,10,11], population [12], and urbanization [13]. The design combines the work of the integrated assessment modeling community and the impacts, adaptation, and vulnerability community into a single framework, facilitating interdisciplinary collaboration. 

Second, we use the World Health Organization’s (WHO) Operational Framework for Building Climate Resilient Health Systems [14]. The framework discusses possible implications for health systems under climate change using six building blocks of health systems: leadership and governance, health workforce, health information systems, essential medical products and technologies, service delivery, and climate and health financing. These building blocks relate to key objectives of health systems, including quality, efficiency, equity, accountability, resilience, and sustainability [15]. Thus, we organize our results around each of the six building blocks and emphasize how building blocks relate to specific health system objectives under each SSP.

The storylines we discuss below have implications for mortality as well as the SDGs. A world with effective health system responses to climate change is likely to face different health challenges than a world where responses are less effective. In the former, health systems are likely to face growing challenges associated with noncommunicable diseases and other health problems associated with aging populations. By contrast, weak responses are likely to be associated with high levels of drivers of childhood mortality, such as diarrheal disease and undernutrition. In addition, these responses have implications for the SDGs, particularly SDG 3 (Good Health and Well-Being). Effective health systems are a critical component of meeting the SDGs in a world of environmental change, particularly targets associated with child mortality, as this mortality is largely preventable with effective public health responses [16]. 

## 2. Methods

In this section, we provide additional details concerning our two frameworks, the SSPs and WHO building blocks, and discuss how we combined these frameworks to expand narratives around health systems and climate change.

**Figure 1:**
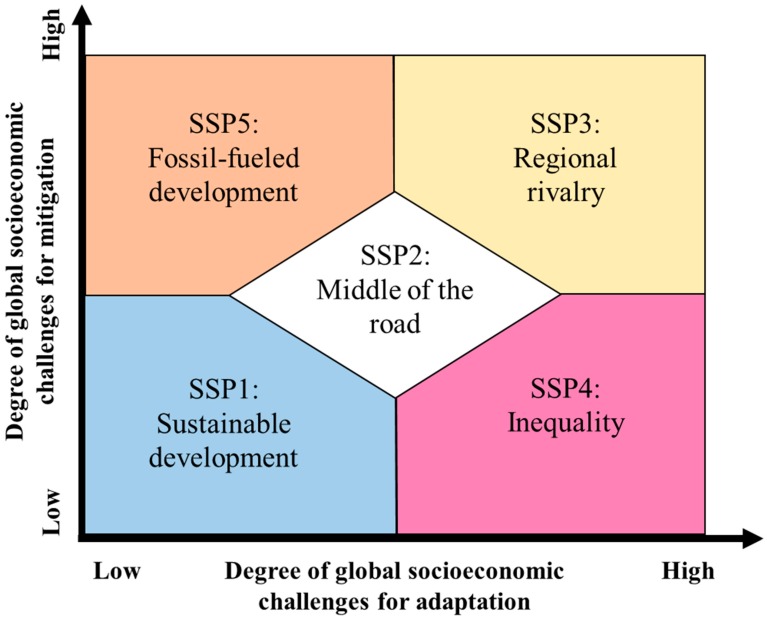
Shared Socioeconomic Pathway (SSP) adaptation and mitigation axes (adapted with permission from O’Neill et al. [17]).

Broadly speaking, SSPs describe potential futures which diverge on axes of increasing challenges to adaptation and mitigation, where each quadrant (high/low challenges to adaptation and mitigation) is represented by an SSP, along with a fifth pathway describing moderate challenges to both (Figure 1) [17]. SSPs range from very optimistic, where governments are able to develop sustainable futures that facilitate adaptation to climate change while largely mitigating its impacts, to very pessimistic, where governments must confront the effects of increasingly severe climate events while lacking the political will or resources to invest in adaptation or mitigation measures, resulting in poor health outcomes [17,18]. 

Scholars have already used the SSPs to make demographic predictions delineated by age group, sex, and educational level [12]. These projections include different assumptions about mortality trends, which extrapolate from current global declines in mortality, but at different rates to account for distinctions between the SSP narratives. Readers can find detailed information concerning how these projections were conducted in [19]. While we anticipate that global mortality will continue to decline, as climate change becomes an even greater threat to global health, we project that an increasing share of variability between countries in mortality outcomes will be explained by health system responses to climate change, which serves as our rationale for expanding the SSP narratives to include additional information on health system effects. We briefly discuss each SSP to provide context for our analyses below.

SSP1 represents a sustainable future, where the world experiences low challenges to adaptation and mitigation [20]. Countries rapidly develop sustainable technologies that result in improved quality of life for all citizens while mitigating further damage to the climate. In addition, development in low/middle-income countries results in improved living standards, leading to an increasingly narrow gap in living standards between low/middle-income countries and high-income countries. High-income countries actively cooperate with and support low/middle-income countries, developing partnerships and funding packages to help facilitate climate adaptation. 

SSP2 represents a future with moderate challenges to adaptation and mitigation [21]. Adoption of sustainable technologies proceeds, although at a less rapid pace than under SSP1. Governments face growing budgetary pressure as climate change hampers the work of various ministries, and more funding is devoted to repairing infrastructure damage from climate and development shocks. In low/middle-income countries, standards of living improve gradually, due in part to continued fossil fuel use as well as by the steady adoption of renewable energy sources. However, pockets of poverty remain entrenched in many of these countries, with additional budgetary pressures resulting from the consequences of extreme weather and climate events. 

SSP3 represents a future with high challenges to adaptation and mitigation [22]. Continued socioeconomic challenges in low/middle-income countries result in minimal improvements to living standards. Governments have little political will and minimal, if any, capital to invest in climate mitigation activities, resulting in increased vulnerability to the effects of climate and development shocks. In high-income countries, moderate levels of income growth along with continued fossil fuel use allow countries to maintain development gains, albeit with minimal improvements over time and inequality within countries. SSP3 depicts a world where inequality and security concerns lead to a resurgence of nationalism, and in some cases, regional cooperation, with very little cooperation between high- and low/middle-income countries on climate change or other issues and limited investment in international coordinating organizations. 

SSP4 represents a future with high challenges to adaptation and low challenges to mitigation [23]. While overlapping in many respects with SSP3, SSP4 is distinguished by very high levels of inequality within and between countries, where capital and political power is increasingly concentrated in a global elite, leaving low-income countries and poorer residents of high/middle-income countries behind. While the use of renewable energy technologies spreads rapidly, particularly in wealthier countries, poorer populations in countries at all income levels experience significant challenges to adaptation, resulting in a growing divergence of demographic, economic, and health outcomes within and between countries. 

SSP5 represents a future with low challenges to adaptation and high challenges to mitigation [24]. While overlapping in many respects with SSP1, SSP5 is distinguished by challenges posed by increasingly severe climate and development shocks, which, despite careful planning efforts, require substantial resources to mitigate. While development outcomes are strong under this pathway, they are not as strong as in SSP1, partly because of the need to devote significant resources to coping with the effects of shocks, as well as the lack of health co-benefits from mitigation activities. 

The WHO Operational Framework for Building Climate Resilient Health Systems was developed to illustrate how various components of health systems are affected by climate change and provide guidance to practitioners and scholars on actions that can strengthen health system resilience to climate change impacts. We believe this is the most comprehensive framework that has been developed to date on climate change and health systems. Moreover, it is particularly useful because it is designed to complement other guidance released for practitioners from the WHO (e.g., [25]) and it provides measurable outputs and indicators that can be used to assess progress in health system adaptation. 

We use the qualitative descriptions provided in SSP background papers, as well as the building blocks and suggested outputs in the WHO framework to illustrate how health system responses are likely to vary under different socioeconomic development pathways. In particular, we take the WHO recommendations as indicative of effective responses, and in general, assume that these will be followed in SSP1 and SSP5. When discussing responses under other SSPs, we assume that responses will be suboptimal based on the WHO framework, in order to draw conclusions about how health systems will less effectively respond to climate change challenges. 

## 3. Results

**Figure 2:**
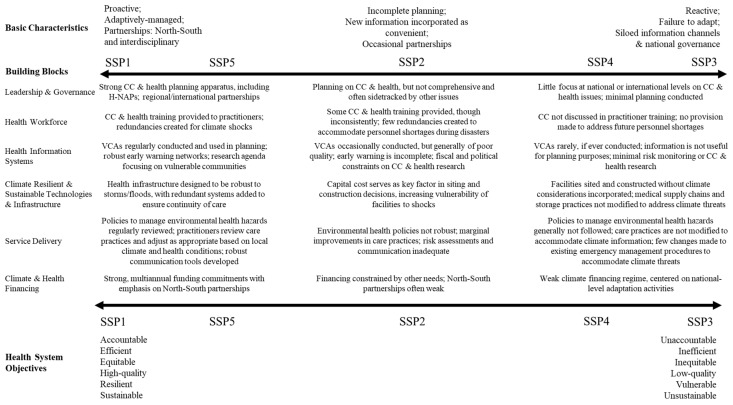
Differences in building blocks across SSPs. Basic characteristics at the top highlight overall differences in management approaches between SSPs. Health system objectives highlights how well each SSP is likely to meet the health system objectives outlined in [15], with SSP1 and SSP3 at the extremes, and SSP2, SSP4, and SSP5 displaying intermediate likelihoods of achieving these objectives.

We discuss the relationship between the SSPs and each building block in turn. The figures in each subsection outline the key differences between SSP1/5, SSP2, and SSP3/4, and also distinguish any differences between SSP1 and SSP5, as well as SSP3 and SSP4, respectively. We group SSP1 and SSP5 together, and SSP3 and SSP4 together, because these SSPs have similar implications for health systems. However, there are distinctions between them. In general, SSP1 and SSP5 are distinguished by the lack of mitigation capacity in the latter, which results in greater damage from climate and development shocks, and creates uncertainty for proactive adaptation. SSP3 and SSP4 are distinguished by high levels of inequality in the latter, resulting in greater intranational variation in responses, with some regions responding relatively adequately to climate and health risks, while poorer regions have few, if any, resources with which to plan. SSP2 represents an intermediate pathway between SSP1/SSP5 and SSP3/SSP4, and is not extensively discussed in this text. For each of the building blocks, health sector responses are projected to be strongest under SSP1/SSP5, and weakest under SSP3/SSP4. Figure 2 presents an overview of our storylines, illustrating the likely effects of each SSP on each of the building blocks examined, as well as the relationship of the SSPs to each other.

### 3.1. Leadership and Governance

High-quality leadership and governance institutions are essential for taking effective actions to address climate change and health linkages. For effective responses to occur, political leaders must recognize the relationships between climate change and health, as well as craft effective policies to address these connections within an iterative risk management framework [26,27,28]. Many governments are already taking such steps, particularly in low- and middle-income countries participating in the United Nations Framework Convention on Climate Change National Adaptation Plan (H-NAP) process, which encourages countries with fewer resources to develop adaptation plans to address medium- and long-term needs [25]. These plans specifically focus on the health-related aspects of climate change adaptation and mitigation, with an aim of improving the quality of health systems and strengthening resilience to the effects of climate change. Developing effective H-NAPs requires effective coordination and collaboration between the health ministry and other government agencies, as well as nongovernmental partners, to ensure that actions taken to address elements of climate and health relationships effectively foster positive health outcomes and do not conflict [29]. 

In addition to national-level actions, effective responses to climate change will require coordination between countries, through regional- and global-level coordinating institutions, including global development banks, the WHO, United Nations Development Program, nongovernmental organizations (NGOs), Red Cross/Red Crescent, and regional consortia of governments such as the European Union. In particular, relationships mediated through these bodies will be critical in helping low/middle-income countries address technical and financial challenges associated with health system adaptation and mitigation efforts. 

**Figure 3:**
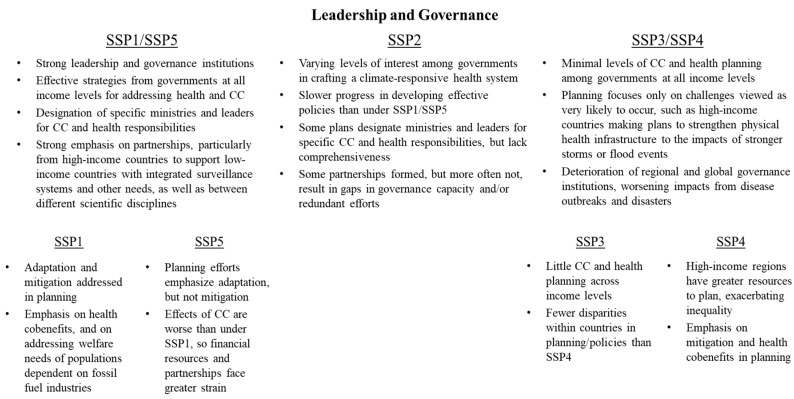
Variation in health system leadership and governance conditions across SSPs.

SSPs differ with respect to the strength of national strategies on climate change and health, as well as the degree to which partnerships are emphasized (Figure 3). Under SSP1/SSP5, clear actions are taken to plan for the health effects of climate and development shocks. Thus, these health systems are most likely to promote accountability and resilience. In high-income countries, this may include an emphasis on responding to epidemics of vector-borne diseases or improving the resilience of health infrastructure to natural disasters. In low/middle-income countries, there is likely to be a greater emphasis on reducing mortality from preventable causes that may be exacerbated by climate change, such as diarrhea or malaria. When appropriate, health authorities under these pathways work closely with nongovernmental organizations to build capacity and improve responses to climate change and health challenges.

Under SSP1 and SSP5, regional and global institutions take strong and decisive measures to prepare and plan for the effects of climate change on health services, and include provisions for technical and financial support to be provided to low/middle-income countries to enhance the adaptation and mitigation capacity of their health services, including through integrated surveillance systems, helping to generate more equitable health outcomes globally. As a result, when infectious disease outbreaks or natural disasters occur as a result of climate change, international responses are swift, well-funded, and effective, resulting in low levels of property damage and deaths. 

Under SSP3 and SSP4, governments take few, if any, steps to plan for the effects of climate variability and change on health services, resulting in less accountable and resilient health systems. Countries that do take steps are likely to be higher-income countries, as these countries have resources that can be invested in planning. However, the steps taken in such countries are meager, with vague and incomplete plans created. Government planning on climate change and health focuses only on challenges viewed as very likely to occur, such as high-income countries making plans to strengthen physical health infrastructure to reduce the impacts of stronger storms or flood events. 

Under these pathways, regional and global institutions are badly under-resourced and largely fail to prepare for climate and health challenges. Some regional blocs may emerge among high-income countries that advance planning and policy around climate and health, however, these activities are not likely to incorporate low/middle-income countries that require the greatest technical and financial support, such as developing and operating surveillance networks. As a result, when disease outbreaks or natural disasters related to climate change occur, international institutions are unable to provide meaningful support to low/middle-income countries. National-level responses from these countries are slow and ineffective, resulting in health systems being overwhelmed by patients, with associated high death tolls. 

### 3.2. Health Workforce

Maintaining a healthy and effective practitioner workforce is a critical challenge for health systems grappling with the potential effects of climate change. Preparing the health workforce for the effects of climate change requires policymakers to think carefully about health worker training, as well as health system capacity. There are several elements of health worker training that policymakers must consider in relation to climate change. First, providing health staff with more extensive training on locally relevant environmental health topics, such as air pollution and water-borne diseases, as well as related topics such as ecology, will be important in ensuring that providers can adequately treat patients [30]. This may include extended modules on environmental health during training for health professionals, and incorporation into continuing education curricula as environmental conditions change. Second, given that conditions associated with climate change are uncertain, risk communication should be a focus of provider training, so that patients can assess the health risks associated with particular environmental conditions and make decisions about their health accordingly [31]. Third, as climate change alters the disease burden in a particular area, workers with particular specialties are more likely to be in demand. This includes infectious disease and environmental health specialists, as well as pediatricians and emergency medicine physicians [32]. Policymakers should use projections about changes in disease patterns to allocate resources for specialist training accordingly. Fourth and finally, health workers are not immune from the health risks of climate change, including climate-related diseases. Health workers may be unable to provide services if their homes or facilities are damaged due to flood or storm events or if an infectious disease outbreak makes travel too risky [33]. Ensuring that there is sufficient redundancy in the health workforce to provide continuity of operations during a disease outbreak or severe weather event is crucial for maintaining public health. Thus, more health workers may need to be trained than in the past and workers may need to be assigned to certain work locations depending on local climate risks. 

**Figure 4:**
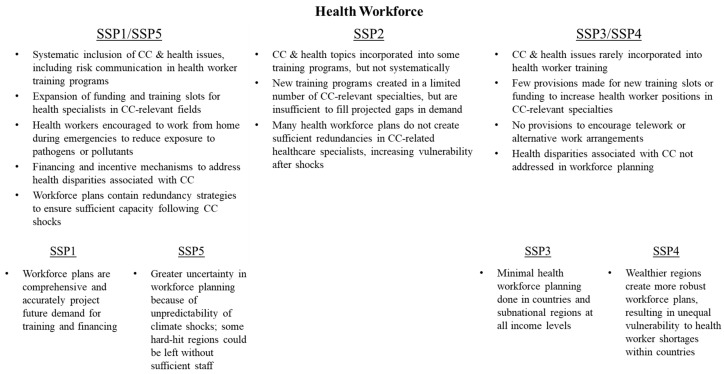
Variation in health workforce conditions across SSPs.

SSPs differ with respect to the strength of workforce training plans and strategies to ensure continuity of services even during climate-related disruptions (Figure 4). Under SSP1/SSP5, government policies incentivize or require information on climate change to be incorporated into the training provided for healthcare providers at all levels, so that health workers can appropriately respond to climate risks, resulting in improved quality of services. Moreover, workforce plans also contain redundancy and resiliency strategies so that in the event of losses of personnel, health infrastructure, or both due to climate-related events, there is sufficient reserve capacity within health services to meet immediate needs. Health workforce plans also develop financing and incentive mechanisms to address health disparities and inequities associated with climate change and ensure that health workers are available and can be placed in underserved areas, as some communities are likely to face greater risks than others. 

By contrast, under SSP3/SSP4, governments provide few, if any, policies to encourage educational institutions to train healthcare workers on climate change or related topics such as risk communication. While individual schools may incorporate such material into their curricula, it is not done systematically nor thoroughly, resulting in lower service quality. Given the high burdens associated with short-term climate change adaptation and mitigation, few governments make provisions for expanding health workforce capacity in response to climate change or for ensuring reserve capacity of staff in the event of natural disasters, ultimately resulting in acute shortages of personnel in critical specialties. 

### 3.3. Health Information Systems

Policymakers and other stakeholders require accurate, timely information on health and health system conditions in order to facilitate effective policymaking activities. The WHO Operational Framework references three key areas where health information systems must be developed: vulnerability, capacity, and adaptation assessments (VCAs), integrated early warning and risk monitoring, and climate and health research. 

VCAs measure potential hazards associated with climate change, as well as the capacity of systems to respond to these challenges. When applied to health, VCAs examine staffing in particular specialties, service quality, physical health infrastructure, and health worker training capabilities, allowing policymakers to understand the vulnerabilities and gaps in capacity that may result from climate and development shocks [34]. As data collection efforts are often too incomplete, inconsistent, or inadequate to provide useful insights to policymakers, one important aspect of VCAs is to identify gaps in climate and health indicators, and creating strategies to fill those gaps when appropriate. Moreover, effective VCAs take into account not only projected changes in climate, but also other socioeconomic factors affecting health outcomes to improve long-term accuracy [35]. 

Integrated early warning and risk monitoring systems provide timely, detailed information on current and future environmental indicators that may affect health outcomes and the ability of health systems to provide services. Such metrics include information on temperature, rainfall, pollution, allergens, and water quality, among other indicators [36]. Additionally, they include recent epidemiological surveillance to measure changes in incidence over time of diseases likely to be affected by climate change and mitigation efforts, such as vector- and waterborne diseases, as well as health conditions that may become less prevalent due to mitigation measures, such as respiratory diseases. These systems also incorporate spatial and climatic information to contextualize the effects of climate change on particular conditions. These monitoring systems are coupled with communication networks (early warning systems) designed to alert members of the public when potential environmental hazards may affect public health, as is increasingly being done with heat events in many countries [28,37]. This information allows interested parties to better understand the health risks associated with particular environmental conditions, helping to focus staff time and resources, as well as identify vulnerable populations that may need to be targeted with special outreach efforts. 

Systems to monitor and communicate information about vulnerability must be complemented by robust climate and health research agendas designed to expand and improve the quality of information provided as the effects of climate change vary over time [38]. Robust research programs that include strong North–South linkages are likely to make health responses to climate change more effective in low/middle-income countries. In addition, research agendas should consider particular communities of interest that may experience disproportionate health burdens related to climate change, including indigenous populations [39], women and girls [40], coastal residents [41], and farmers [42], among others. 

**Figure 5:**
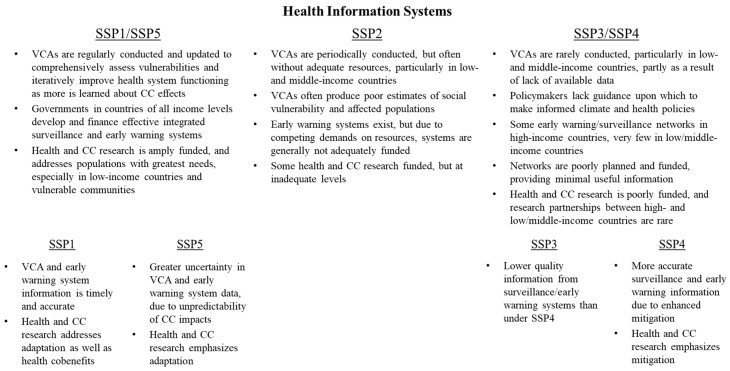
Variation in health information systems across SSPs.

SSPs vary considerably in terms of investments made in knowledge-gathering, resulting in high-quality information under SSP1/SSP5, and lower quality information (and often substantial gaps in knowledge) under SSP3/SSP4 (Figure 5). Under SSP1/SSP5, VCAs are regularly conducted and updated to comprehensively assess vulnerabilities and iteratively improve health system functioning as more is learned about the effects of climate change. Policymakers and health leaders understand likely disease risks, as well as which populations are most vulnerable, and have personnel and procedures to specifically address likely hazards. Additionally, governments in countries of all income levels develop and finance integrated surveillance and early warning systems (where feasible) that rapidly provide a variety of climate-relevant health information to clinicians, researchers, policymakers, and the public. Finally, research programs with ample funding and strong partnerships within and outside governments across regions enhance understanding of climate and health linkages, making responses more timely and effective. Collectively, these efforts are likely to improve health system efficiency as information is used to improve resource allocation. 

By contrast, under SSP3/SSP4, VCAs are rarely conducted, particularly in low/middle-income countries, partly as a result of a lack of available data. As a result, policymakers lack guidance upon which to make informed climate and health policies. Surveillance networks to assess health and climate vulnerabilities are poorly planned and funded in high-income countries, providing little useful information for stakeholders. In low/middle-income countries, these networks are generally not developed due to competing demands on resources, as well as a lack of partnerships with high-income countries. Additionally, research on health and climate change receives minimal funding and government support in high-income countries. The research that occurs emphasizes short-term vulnerabilities to the effects of climate variability. Low/middle-income countries are unable to fund significant climate and health research agendas under these pathways, and receive very little, if any, research funding support from high-income countries. This lack of research and development weakens population health activities, hampering responses to infectious disease outbreaks.

### 3.4. Essential Medical Products and Technologies

Climate change is likely to affect the ability of healthcare systems to keep patients healthy, particularly during periods of extreme weather and infectious disease outbreaks, due to infrastructure damage and medical supply disruptions. Appropriately siting facilities and constructing buildings with weather-resistant materials is essential. Additionally, practitioners must take appropriate steps to ensure that their facilities are prepared to handle various emergencies associated with climate and development shocks, including providing various redundant systems, so that facilities can safely operate in adverse conditions [33,43]. 

Shocks may also disrupt supply chains of essential medical supplies, making it difficult for facilities to deliver care. In particular, there are concerns about the safe transport and storage of temperature-sensitive pharmaceuticals and vaccines, which may be irreparably damaged if improperly stored for too long [44]. Some pharmaceuticals may function differently when taken at high temperatures, leading to unintended outcomes for patients. 

**Figure 6:**
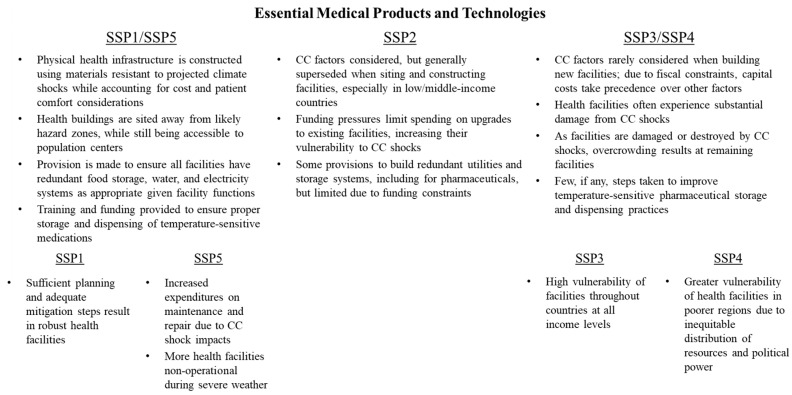
Variation in essential medical products and technologies across SSPs.

SSPs differ in terms of how quickly and robustly physical infrastructure improvements are made to health facilities to ensure they are climate-resilient (Figure 6). Under SSP1/SSP5, governments in countries at all income levels plan physical health infrastructure that is resistant to damage from climate shocks through the use of appropriate materials and siting away from hazardous areas. In cases where health facilities lack redundant systems for food storage, water, and electricity, governments make provisions to provide funding for these systems so that facilities are more resilient during disasters. In addition, health facilities practice proper infection control practices.

Of particular note is that under SSP5, unlike SSP1, an increasing share of health system budgets are likely to be spent on facility repair and upkeep due to severe weather and climate events, leaving fewer funds available for training, staff wages, and patient care. This is especially true in low/middle-income countries, where there is generally higher vulnerability to severe storm and flood events, as well as lower construction standards. This unfortunate combination is likely to result in an increased number of health facilities being non-operational during and after severe weather, leading to overcrowding at facilities that manage to stay open. 

Under SSP3/SSP4, growing fiscal pressures in countries at all income levels make improving the resilience of healthcare infrastructure to climate change an issue of minimal concern. Fiscal strains require that new health facilities are sited primarily on capital cost considerations, even if such facilities are more vulnerable to climate shocks that increase long-term maintenance and operating costs. Few funds are available to retrofit older facilities to be more climate-resilient. As a consequence, major storms and flood events cause catastrophic damage to health facilities, endangering the health and well-being of patients and staff, and thus making recovery more expensive. After these events, some facilities may not be repaired due to the cost, resulting in overcrowding at remaining facilities. Finally, few steps are taken to increase the resilience of medical supply chains to disruptions from climate variability and change, or to improve storage and prescription practices around temperature-sensitive pharmaceuticals. 

### 3.5. Service Delivery

While the planning processes discussed above are critical towards developing a climate-resilient health system, they will matter little if governments fail to effectively deliver services. Towards that end, WHO guidance details three areas where governments must innovate to effectively deliver services: managing environmental determinants of health, creating climate-informed health programs, and developing robust emergency preparedness and management processes. 

After decisions are made about climate change and health responses, ensuring that effective policies to control environmental determinants of health are enacted and maintained will be critical to providing effective services. Using the surveillance networks described above, health systems must work closely with agencies that monitor food, water, air quality, and waste. When monitoring data indicate dangerous conditions, health authorities should take proactive programmatic and policy steps to reduce harmful discharges and exposure to particular contaminants. As likelihoods of disease outbreaks or property damage vary due to changes in climatic conditions, regulations should be updated to reflect current understandings of risks [45]. 

Health practitioners may also need to rethink how services are delivered to accommodate a world with climate change. Using the aforementioned surveillance systems and response plans, policymakers and health professionals must adjust their service delivery strategies to accommodate the needs of patients at risk of climate change. For instance, individuals particularly susceptible to allergens should be followed up more regularly by health staff as allergen loads increase in the environment [46]. As climate change can adversely affect mental health conditions [47], mental health practitioners should have plans in place to deal with increased caseloads during and after disasters, as well as have the ability to provide services in non-clinic settings, as vulnerable populations may have difficulty accessing facilities. 

In addition, other aspects of healthcare delivery are likely to change in the future, affecting the baseline health of populations and health system capacity. For instance, many high-income countries have universal healthcare systems, which generally result in better population health outcomes than systems where only a portion of the population has medical insurance [48]. Under SDG 3, Target 3.8 stipulates that all governments strive to achieve universal health coverage by 2030. If this goal is met, then population health is likely to be much improved, provided that other aspects of health infrastructure and workforce capacity are also addressed. As a result, it is less likely that illnesses or injuries from climate-related events will result in severe morbidity or mortality, particularly in low- and middle-income countries. 

Finally, in order to deliver health services continuously throughout climate-related events, governments must craft effective emergency management plans and adopt effective practices, including clearly communicating health risks to members of the public as events unfold. Provision of robust communications equipment and redundant medical supplies should be part of any plan. Plans should be developed at the national and facility levels, and should be based on input from appropriate stakeholders, as well as incorporate up-to-date assessments of risk. The effectiveness of emergency management personnel in communicating health risks is likely to vary based on education levels within communities, which also vary by SSP. Greater efforts and simpler messaging strategies, co-produced with communities of interest, will likely be needed in areas with lower levels of education, so that instructions from health professionals are more likely to be followed [49]. 

**Figure 7:**
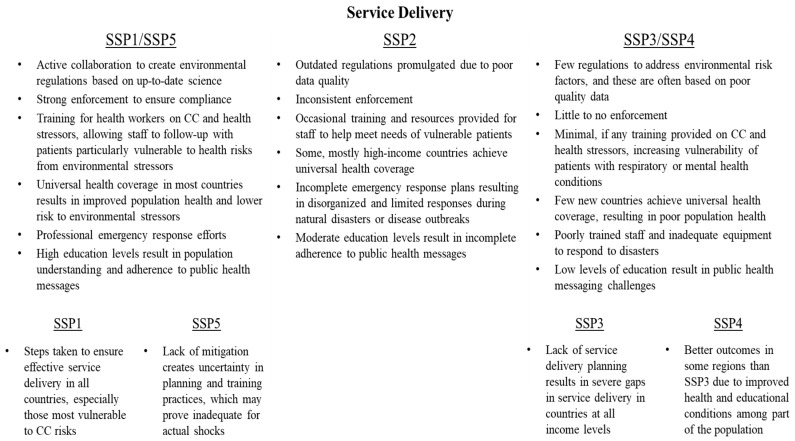
Variation in health system service delivery conditions across SSPs.

SSPs vary in the likelihood that the information and planning discussed above is incorporated into programs (Figure 7). Under SSP1/SSP5, health systems actively collaborate with other agencies to ensure that environmental regulations are based on up-to-date scientific understandings, and that rules are effectively enforced to ensure compliance. Effective planning and training efforts result in health professionals of all specialties capable of delivering high-quality climate-adapted health services, ensuring that patients receive care tailored to changes in environmental conditions, as well as care delivered during natural disasters with minimal interruption. In addition, under SSP1/SSP5, universal health coverage is realized in the vast majority of countries around the world, resulting in substantial improvements to baseline health indicators. Moreover, high education levels under these pathways help ensure that public health messages are understood, and members of the public take proactive steps to protect their health before, during, and following climate shocks.

Under SSP3/SSP4, lack of surveillance, as well as a reactive approach to policymaking, result in increasingly polluted food, water, and air, and substantial health risks associated with poor quality waste disposal. Regulations, if they do exist, are rarely enforced and fail to prevent frequent pollution discharges that result in large-scale disease outbreaks. Few health professionals, except those serving the social elite, change their practices as a result of environmental surveillance or similar data, if these data exist. Moreover, population health remains poor in many low- and middle-income countries as few additional countries achieve universal health coverage under these pathways. Because of poor population health conditions and the poor quality of health services provided, climate shocks remain particularly deadly. Finally, emergency preparedness efforts are minimal, with governments reacting to disasters with incomplete information, as well as poorly trained and equipped staff, leading to ineffective responses. In addition, low levels of education make it substantially more difficult to communicate risks to members of the public during disasters, leading many individuals to continue to engage in behaviors (such as using contaminated water or continuing to inhabit an area at risk of storm damage) that place them at high risk of morbidity or mortality.

### 3.6. Climate and Health Financing

Prioritizing and securing sufficient financing is likely to serve as a major obstacle to adapting health systems to challenges associated with climate change. A key element of financing policy is ensuring that climate and health system adaptation is provided a line item on health services and donor budgets, ensuring that there is continued attention of policymakers to climate and health issues and that funding is likely to be sustained over time. Additionally, a robust funding system also includes resources to sustain partnerships between the Global North and South, helping to improve adaptation capacity in under-resourced countries. Such funds are likely to flow through one of the already-existing international climate funds (Green Climate Fund, Climate Investment Funds, Adaptation Fund, etc.) that are set up to finance adaptation projects in low/middle-income countries. It will be important in a climate-resilient future that these and similar institutions remain intact to ensure the stability of funding streams and partnerships over the coming decades. 

**Figure 8:**
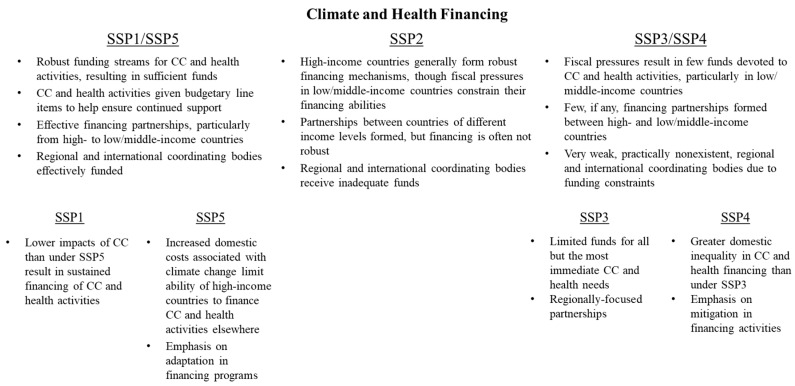
Variation in climate and health financing conditions across SSPs.

SSPs differ in regard to the amount of funds provided for health system adaptation, as well as the degree to which partnerships and collaboration are emphasized in funding processes (Figure 8). In SSP1/SSP5, such prioritization of climate and health funding is likely to occur throughout health systems at all organizational levels, generally resulting in increasing funds made available for necessary climate and health purposes, helping to ensure the sustainability of climate and health activities. National governments work closely with regional and international intergovernmental organizations, as well as private donors, to pool funds to address joint challenges. Under these pathways, governments work with international climate funds to prioritize spending on climate and health adaptation, with a particular focus on vulnerable populations, helping to reduce global mortality disparities. 

Under SSP3/SSP4, high-income countries are most likely to fund climate and health efforts, albeit at insufficient levels. Low/middle-income countries, which are likely to experience more severe fiscal pressures due to other development priorities, are unlikely to devote sufficient funds to these efforts, resulting in poorer population health outcomes. Because of a lack of mitigation efforts, demand for adaptation funding grows, especially in low/middle-income countries, even as the supply of these funds from high-income countries is limited. This tension places substantial strain on intergovernmental relationships, and makes effective international partnerships to address climate and health challenges virtually impossible. Moreover, donor organizations created to finance climate change adaptation efforts in low/middle-income countries receive minimal support from high-income countries. Under SSP3, the funding and partnerships that do materialize are largely regionally concentrated, with high-income countries supporting the preparedness efforts of nearby countries as a way of bolstering regional security and reducing the possibility of refugee flows. Under SSP4, because of pervasive inequality, financing partnerships that are formed are generally not designed to reach the most vulnerable populations domestically or abroad, resulting in worsened health disparities.

## 4. Discussion

Our findings have substantial implications for health policymakers going forward, given the myriad ways in which health and climate change intersect. Below, we briefly discuss two implications of the discussion above on the SDGs and on the global distribution of mortality. 

**Figure 9:**
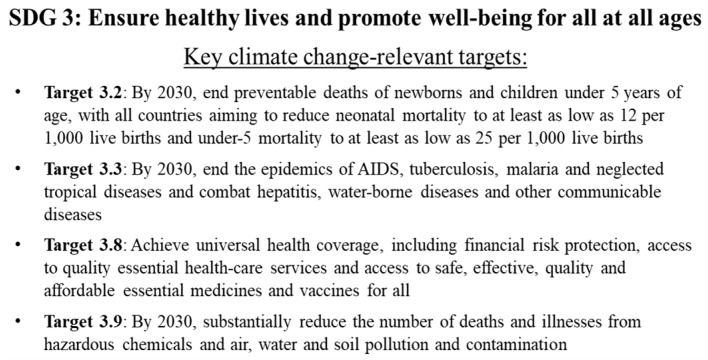
Sustainable Development Goal (SDG) 3 and key climate-relevant targets.

The health trajectory of each SSP has implications for achieving the SDGs, particularly SDG 3, which centers on health. While we do not include quantitative projections, and cannot say whether each target will definitively be met under a particular SSP, we can suggest that based on the health-related challenges experienced under each SSP, some SSPs are more likely to meet SDG targets than others. While all SDG 3 targets have a connection to climate change, there are several targets in particular that are likely to be particularly affected by health system responses to climate change outlined above (Figure 9). 

Under SSP1/SSP5, most of the SDG 3 targets appear achievable, including Target 3.2 to end preventable childhood mortality, and Target 3.3 to end the AIDS, TB, and malaria epidemics, particularly in light of the fact that health systems under these SSPs are likely to deliver higher-quality and more equitable services A key difference between SSP1 and SSP5 is that SDG 3 targets affected by environmental variables sensitive to fossil fuel development, such as Target 3.9 to reduce deaths and illnesses from hazardous chemicals and air, water, and soil pollution, are less likely to be met under SSP5, given continued fossil fuel development under this pathway. However, projections suggest that air pollution levels for most contaminants are lower under SSP5 than under other SSPs except for SSP1, implying that some of these fossil fuel-affected targets may also be achievable under SSP5 [50]. 

Under SSP2, it is likely that governments will come closer to meeting Goal 3 targets than under SSP3 or SSP4, although given the lack of strong and deliberate steps to improve health care in SSP2, it is likely that these reforms will be insufficient to actually meet the targets. By contrast, most of the targets under SDG 3 are unlikely to be met under SSP3 or SSP4 given the failure of governments in these pathways to sufficiently adapt health systems to climate challenges. Other SDGs that have clear implications for health, such as SDG 2 (Zero Hunger) and SDG 6 (Clean Water and Sanitation), are likely to be affected in similar ways as SDG 3, where achievement is most likely under SSP1/SSP5 due to increases in GDP and sustainable development policies, and least likely under SSP3/SSP4. 

Changes in healthcare systems under different SSPs are likely to alter how mortality occurs around the world as climate change becomes a larger driver of global health outcomes. Because of improvements to health service delivery under SSP1/SSP5, fewer deaths will be associated with preventable causes, such as a reduction in deaths associated with causes such as undernutrition, malaria, and diarrheal diseases [3]. Health and development interventions, such as increased vaccination rates, as well as growing access to mosquito nets and safe drinking water supplies make these conditions less prevalent. While occasional intense storms or flood events may produce temporary upticks in water- or vector-borne diseases, strong health systems with resilient infrastructure and well-trained staff prevent these incidents from developing into epidemics. 

Although baseline rates of mortality attributable to climate-related causes are higher in low/middle-income countries, strong partnerships established under SSP1 and SSP5 result in these countries eventually catching up with high-income countries in low rates of communicable disease. As a result, in the coming decades, a substantially greater fraction of mortality is associated with cancer and cardiovascular disease in virtually all countries around the world. Under SSP2, mortality from preventable conditions falls, but more slowly than under SSP1 and SSP5, as health and climate interventions under this pathway are less robust. In low/middle-income countries, health outcomes improve, although government resources are sometimes stretched thinly, as health systems struggle to balance the health needs of a growing middle class increasingly experiencing chronic conditions with the challenges of poorer populations that continue to experience preventable diseases exacerbated by climate change. 

Under SSP3/SSP4, a failure to mitigate the effects of climate change and invest in health services results in slower declines in mortality rates in countries at all income levels. In high-income countries, mortality from respiratory diseases increases due to fossil fuel use. In some cases, poor public health preparation and significant climate shocks precipitate the return of vector-borne diseases to places where they had been previously eradicated, such as the American South. Thus, increased deaths from malaria and in some cases, diarrheal disease, displace deaths that would have occurred due to cancer or cardiovascular conditions. In low/middle-income countries, the mortality transition stalls nearly completely, with undernutrition, malaria, and diarrheal diseases continuing to serve as major causes of death, particularly among young children. Lack of effective early warning systems and low adaptation capacity due to poverty results in high mortality from weather events, particularly heat waves and floods. However, the stochastic nature of these events results in substantial interannual variability in causes of death, with cataclysmic levels of mortality from heat waves and floods experienced in some years, while in other years, few, if any, deaths result from these causes. Specifically, under SSP4, a small, but wealthy elite experiences improved quality of life and a shift towards diseases of affluence, while the poorer majority of the population experiences continued vulnerability to malnutrition, diarrhea, and malaria. Mitigation measures such as the promotion of active transport and clean cookstoves under this pathway result in improved overall health and reductions in deaths from respiratory diseases.

Finally, readers should keep in mind that the discussion above relates to a world where all countries follow a single SSP. It is possible that some high-income countries have different development trajectories than their low- and middle-income counterparts, or that there is strong heterogeneity within country income groupings. In that event, the analysis presented above may become more complicated, with different national or regional outcomes depending on development pathways chosen. Further, as shown in the preceding discussion, there are likely to be interactions across various drivers within a SSP, thereby increasing the complexity.

## 5. Conclusions

We outline some of the major ways through which health systems are likely to be affected under different SSPs, illustrating that very different health system responses are possible under each pathway, with substantial implications for population health and the mortality distribution. Further research should seek to explore different cases at a variety of scales to examine how different health systems are coping with health and climate change challenges, and to what extent best practices are emerging in this field which can be promoted and adapted by other health systems facing similar challenges. Moreover, efforts to quantify the change in the mortality distribution under different SSPs and by geographic region will provide essential information for policymakers seeking to understand how to best allocate health resources in a world stressed by climate change. Such quantifications also should include temporal considerations, such as what could be the range of characteristics of health systems in the 2030s, when the increase in global mean surface temperature will be about 1.5° above pre-industrial temperatures. These quantifications would inform modeling of the health risks of climate change, to support policy development within and outside health systems that promotes sustainable and resilient health systems in a changing climate.
